# The effects of a music and singing intervention during pregnancy on maternal well-being and mother–infant bonding: a randomised, controlled study

**DOI:** 10.1007/s00404-020-05727-8

**Published:** 2020-08-10

**Authors:** Verena Wulff, Philip Hepp, Oliver T. Wolf, Percy Balan, Carsten Hagenbeck, Tanja Fehm, Nora K. Schaal

**Affiliations:** 1 Department of Experimental Psychology, Heinrich-Heine-University Düsseldorf, Universitätsstraße 1, 40225 Düsseldorf, Germany; 2 Clinic for Gynecology and Obstetrics, University Clinic, Augsburg, Germany; 3 Clinic for Gynecology and Obstetrics, HELIOS University Clinic, University Witten/Herdecke, Wuppertal, Germany; 4 Department of Cognitive Psychology, Institute of Cognitive Neuroscience, Faculty of Psychology, Ruhr-University, Bochum, Germany; 5 Clinic for Gynecology and Obstetrics, Heinrich-Heine-University Düsseldorf, Düsseldorf, Germany

**Keywords:** Pregnancy, Music, Singing, Mother–infant bonding, Maternal well-being

## Abstract

**Purpose:**

Stress and impaired mother–infant bonding during pregnancy can lead to adverse effects for the expectant mother and the unborn child. The present study investigates whether a prenatal music and singing intervention can improve maternal well-being as well as mother–infant bonding.

**Methods:**

A total of 172 pregnant women took part in this prospective, randomised, three-armed (music, singing or control group) study. Depressive symptoms, self-efficacy, maternal well-being and mother–infant bonding were assessed with visual analogue scales and questionnaires before the intervention phase (30th week of gestation) and afterwards (36th week of gestation). Additionally, immediate changes regarding experienced stress and mood from before until after the music and singing interventions were explored with questionnaires as well as saliva samples (for cortisol, alpha-amylase and oxytocin determination).

**Results:**

Regarding immediate effects, both interventions showed positive effects on the emotional state, stress (cortisol) and bonding (oxytocin). Additionally, the singing group showed a larger reduction in cortisol and a larger improvement in valence than the music group. Looking at more prolonged effects, significant effects on general self-efficacy and perceived closeness to the unborn child (measured with a visual analogue scale) were found. No significant effects were revealed for the mother–infant bonding questionnaire and for depressive symptoms.

**Conclusion:**

In the present study, promising effects of music and in particular singing on maternal well-being and perceived closeness during pregnancy appeared. Prenatal music and singing interventions could be an easy to implement and effective addition to improve mood and well-being of the expectant mother and support mother-infant bonding.

**Trial registration number:**

DRKS00012822, date of registration: 17.10.2017

**Electronic supplementary material:**

The online version of this article (10.1007/s00404-020-05727-8) contains supplementary material, which is available to authorized users.

## Introduction

Pregnancy is a very special and important time in a woman´s life, in which the mother-to-be prepares herself for her new role as a mother. This time is usually filled with feelings of excitement and joy; however, stress and negative emotions during pregnancy are also very common. In the last decade, an increasing amount of studies have looked at the impact of stress during pregnancy on the mother-to-be and the baby’s development [51]. Several studies have shown that higher stress is associated with higher rates of spontaneous preterm labour and lower birthweight [53, 57, 76, 77]. Gestational stress and anxiety can also have a negative impact on postpartum maternal well-being such as postpartum depression [70]. There is also evidence that stress negatively impacts the development of the (unborn) child regarding cognitive and motor development [7, 37, 72, 81 and temperamental and behavioural measures [29].

Another factor to consider, which is important not only during pregnancy but also postnatally with regard to mothers’ mental health and the child’s development, is mother–infant bonding. Studies have shown that mother–infant bonding increases over the course of gestation, especially when women feel the first movements of the fetus [68, 71]. It has also been shown that strong mother–infant attachment is associated with positive effects on maternal mental health and depressive symptoms [69]. Moreover, it has been shown that insecure attachment is related to higher anxiety in children [11] and secure attachment is related to better emotion regulation abilities [6]. Additionally, a study has also revealed that maternal stress and mental health problems during pregnancy and after birth negatively impact mother-infant bonding [71]. Thus, it is important to investigate whether interventions can reduce stress and anxiety and improve mother-infant bonding during pregnancy. To this end, the present study investigates the effect of a music and singing intervention during pregnancy on maternal well-being and mother–infant bonding.

In an increasing body of literature the positive effect of music on mood, well-being and health has been shown. A meta-analysis of Pelletier [65] concluded that music leads to decreased arousal levels and a faster recovery from stress. In particular, positive effects of music are reported in clinical settings [35] where reduced anxiety, postoperative pain and use of analgesia were reported as results of different kinds of music interventions. In the context of obstetrics it has been shown that music can be an effective intervention to reduce stress and anxiety during anxiety-inducing situations. For example, music that is presented during a caesarean section led to lower anxiety measured by subjective (questionnaires) and objective (salivary cortisol and heart rate) parameters [33] and in women awaiting an amniocentesis, listening to music resulted in less reported anxiety and lower cortisol levels compared to control groups [82]. In general, stress leads—amongst other things—to an increase of cortisol levels that is caused by an activation of the hypothalamus–pituitary–adrenal axis through the limbic system [42]. Due to that, cortisol is an often used marker for the physiological stress response [31]. Another biomarker that is used for measuring stress is alpha-amylase that increases due to stimulation of the sympathetic nervous system as a consequence of stress [59]. Both biological markers can be easily measured in salivary which is well-established and therefore often used in experimental stress-studies [31, 60, 79].

Several reviews highlight that music interventions during pregnancy also have a general anxiety reducing effect [50, 80, 84]. Moreover, studies have shown that music interventions among pregnant women have even specific positive effects in risk groups. Apart from the conventional treatment in case of pregnancy-induced hypertension, women that received music therapy in addition to the standard treatment showed significant lower blood pressure, less subjective anxiety and depression scores compared to a control group [8]. Furthermore positive effects of daily listening to music were shown in a study with 88 pregnant women who suffered from poor sleep quality. After four consecutive weeks of music listening, the experimental group showed significant better values in different sleep quality indices compared to a control group [75]. In general, music seems to have beneficial effects on the psychological health like perceived stress, anxiety and depressive symptoms during pregnancy [10].

As a special form of active music making, also the effects of singing on psychological and physiological parameters have been investigated in recent years. Kang, Scholp, and Jiang [39] revealed in their review, that singing interventions can lead to an improved lung function, an increased heart rate variability and lower blood pressure. Due to the active part of breathing and using the own voice as an instrument, it has been shown that singing has a positive impact on the cardiorespiratory system [4, 83]. Beside the physiological effects, singing can also have a positive influence on several factors like psychological health and well-being [24]. Furthermore, it has been shown that singing can also have communicative and interactive components when singing together with others for example in a choir. Kreutz [45] investigated the effects of a singing session on self-reported and physiological measures and showed an improvement in psychological well-being and a reduction in cortisol levels after the singing interventions. Moreover, a study that explored the influence of music and singing after childbirth on maternal well-being and mother-infant bonding revealed improvements in well-being, self-reported mother-infant bond as well as in self-esteem and depressive symptoms [17]. In addition to these effects that are based on subjective measurements it has been shown that music and singing lessons can lead to higher levels of oxytocin, a hormone that is typically associated with social behaviour [27, 41, 54, 62] and with mother–infant relationship [22]. A study in the field of obstetrics revealed that higher levels of oxytocin measured during pregnancy and after birth were associated with better maternal behaviour [21]. Therefore, it is plausible to measure oxytocin concentrations when exploring effects regarding mother-infant attachment.

Until now, only a few studies have investigated the effects of active singing on maternal well-being during pregnancy [9, 67]. Carolan et al. [9] conducted a qualitative study on the effects of repeated singing sessions which were part of antenatal classes on stress levels. In depth interviews with six pregnant women revealed that singing lullabies led to higher reported relaxation and improved perceived connection to the unborn child. To the best of our knowledge, the only known randomised controlled trial study up to now was conducted by Persico et al. [67] and examined the influence of prenatal singing during pregnancy on mother–infant bonding, newbornsʹ crying behaviour and maternal stress in 156 women. Participating women were randomised into two groups. The control group received standard antenatal classes and the experimental group took part in antenatal classes which included singing elements. The women participated in 14 sessions. The gestational age of the women was around 24 weeks at the beginning of the intervention period. In addition to the starting point, different measurements were conducted at 36th week of gestation, 48 h after birth, 1 and 2 months after birth and the last time of measurement took place 3 months after birth. Effects in favour of the singing group were found for the postnatal period only in which neonatal crying episodes and perceived maternal stress were reduced in the intervention group. Taken together, the studies by Carolan et al. [9] and Persico et al. [67] as well as the results reported after birth [17, 56] highlight that maternal singing can have positive effects on relaxation, well-being and mother-infant bonding.

In light of the fact that to the best of our knowledge no study to date has compared the effects of music and singing during pregnancy in one study, this study for the first time investigated the influence of active singing in comparison to passive music listening during pregnancy on mother-infant bonding, well-being, depressive symptoms and self-efficacy. In line with previous research highlighting the positive impact of music and singing on mood, stress related factors and bonding [8, 17, 48, 65, 67] we expected that the interventions (singing and music) will lead to improved mood, higher mother-infant bonding, reduced stress, and depressive symptoms, and higher self-efficacy. The factors were assessed with validated questionnaires and salivary samples at different time points (namely in the 30th and 36th gestational week and, in the intervention groups, before and after the music and singing lessons). The improvements were expected for the used subjective measurements (questionnaires) during the intervention as well as in the short-term period during the last trimester of pregnancy. In addition, improvements were expected for the biological markers where a reduction of salivary cortisol and alpha-amylase and an increase in salivary oxytocin were expected from pre to post intervention. In the context of the active part of singing, additional and larger effects were anticipated for the singing in contrast to the music group.

## Methods

### Participants

**Fig. 1:**
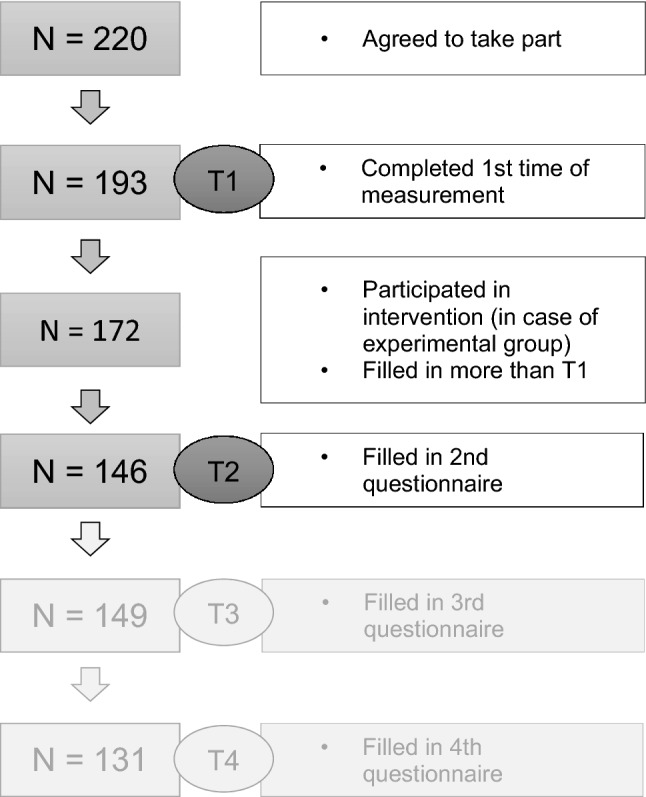
Sample sizes at the different stages of the total project. The focus of the present manuscript lies on the effects of the intervention during pregnancy and therefore only T1 and T2 are considered

Initially, 743 pregnant women at the Clinic for Gynaecology and Obstetrics at the University Hospital Duesseldorf were screened and offered participation between November 2017 and January 2019 and 220 of them agreed to take part. The women were 18–42 years (*M* = 34.03, *SD* = 3.89) old and at the time of recruitment participants had a gestational age between 24 + 0 and 35 + 6. Only women aged above 18 years, without serious comorbidities or pregnancy risks and with sufficient knowledge of the German language were eligible to take part. All participants gave informed written consent prior to the start of the study and were afterwards randomly assigned to one of the three study groups (singing, music, control). The final sample consisted of 172 participants, because 27 women did not fill out the first questionnaire although they gave their consent to participate and 21 women did not participate in the interventions. The sample of participants varied over the two times of measurement due to alternating inaccessibility over time. For an overview of the sample size see Fig. 1. The total study included two additional times of measurement after birth (48 h (T3) and 8 weeks (T4) after birth) that are not included in this paper as the present paper concentrates on the prenatal effects of the music and singing intervention. To calculate the necessary sample size, the program G*Power [19] was used. The power analysis was based on the primary outcome mother-infant bonding. A low to medium effect size (*d* = 0.2) was expected for the overall study project that contained three groups and four times of measurement. With a power of 80% and an alpha-error of 0.05, the required sample size is 156 (52 per group).

### Material

#### Self-assessment manikin

To measure the participants´ emotional state before and after the music and singing interventions, the Self-Assessment Manikin (*SAM* [5]) was used. Participants were asked to rate their actual affective state on the three dimensions valence, arousal and dominance via visual figures that represent each dimension. Every affective state can be rated on a 9-point Likert scale with five figures and the possibility of four intermediate ratings between the figures. The affective expressions of the figures range from “pleasant” to “unpleasant” in the valence dimension, from “excited” to “calm” in the arousal rating and from “dependent” to “independent” in the dominance rating. For each dimension, the score varies between zero and five. Higher scores indicate less pleasure, less arousal and higher dominance.

#### Saliva samples for salivary cortisol, alpha-amylase and oxytocin detection

Saliva samples were taken before and after the music and singing interventions to determine cortisol, alpha-amylase and oxytocin levels. For saliva collection Salivettes (Sarstedt, Germany) were used. Participants insalivated the cotton swabs for at least 30 s and the samples were then stored at – 18 °C until further analysis. Cortisol and alpha-amlyase samples were analysed in the laboratory of the DresdenLAB (Dresden, Germany) by using an immunoassay and a quantitative enzyme [74] whereas oxytocin was determined by RIAgnosis (Munich, Germany) with a radioimmunoassay [15].

#### State-trait-anxiety

Anxiety was measured with the State-Trait-Anxiety Inventory [46]. With two different questionnaires, each consisting of 20 questions, the temporary feeling of anxiety during a particular moment (State) and the general tendency towards anxiety (Trait) can be detected. Participants rate their answers to different statements on a 4-point Likert scale from “almost never” to “almost always”. Only the STAI-Trait questionnaire was used to control for possible differences in trait anxiety between groups. For analysis, the 20 items were added to an overall score (possible range 20–80). A higher score indicates higher trait anxiety.

#### Visual analogue scale

To measure the subjective value of the perceived closeness to the baby, a visual analogue scale (VAS) was used. The scale consisted of a 10-cm line and the women were asked to mark the point on the line which corresponds to their actual feeling. Women were asked the question “How close do you feel to your baby?” and the anchors of the line were “no closeness to the baby” on the left end and “maximum closeness of the baby” on the right end. For analysis, the distance from the left end of the scale was measured in cm with a higher score indicating a higher perceived closeness to the child.

#### Maternal antenatal attachment

To measure the prenatal attachment of the expectant mother towards the baby, the German version of the Maternal Antenatal Attachment Scale (MAAS; Beetz and Behringer, 2006. “German translation of the Maternal Antenatal Attachment Scale [12]” Unpublished translation.) was used. The MAAS is a valid and reliable instrument to assess the presence and strength of maternal–fetal attachment [12, 13]. The questionnaire consists of 19 items and measures the two dimensions “intensity or time spent in attachment mode” and “quality of attachment”. Participants were asked to rate each item on a 5-point Likert Scale and for further analysis, cumulative scores were calculated for both dimensions where the score of “intensity” (eight items) ranges from 8 to 40 and the score of “quality” (11 items) ranges from 11 to 55. In both dimensions, higher scores indicate higher or stronger attachment.

#### General self-efficacy

General self-efficacy is a construct that reflects the evaluation of the personal competence to cope with difficulties and difficult situations [34]. In the present study, the concept was measured with the General Self-Efficacy Scale (*Allgemeine Selbstwirksamkeit Kurzskala,* ASKU; [1]). The scale consists of three items and participants rate their answers on a 5-point Likert scale (from “does not apply at all” to “applies completely”). For further analysis, a mean score of the three items was calculated that reflects the general (subjective) expectancy of self-efficacy with higher scores indicating higher self-efficacy.

#### Pre- and postnatal depression

The Edinburgh Postnatal Depression Scale (EPDS) is a 10-item questionnaire for the screening of depressive symptoms [14]. In the present study, the validated German version of the EPDS was used [2]. Participants were asked to rate their feelings during the last 7 days on a 4-point Likert scale with values from 0 to 3. A cumulative score that depicts the strength of depressive symptoms, is calculated. The score can range from 0 to 30 and a higher score indicates a higher probability of having a depression. The questionnaire can be used postpartum as well as during pregnancy [3].

### Interventions

Participants in the music group joined one music session with up to three other women between the first time of measurement (T1) and the 34th gestational week. In the session, they practised relaxation through passive music listening and received instructions on how to listen and relax to music at home. The lessons started weekly at 15:30 h and lasted approximately 30 min. After arriving, the women were welcomed and asked to give the first saliva samples and to fill out the first questionnaire about their subjective well-being (SAM). After this was completed, the participants were invited to sit down comfortable on gymnastic balls for the music session. An explanation about the goal of the music intervention—to consciously take time to listen to the music and to try to relax while listening—was given. Participants were asked to continue the music intervention on a daily basis at home for at least 10–15 min per day until the time of birth. It was emphasised that it would be desirable to perform the intervention without any disturbances and to try to integrate the intervention as a daily routine. Every participant received a CD with classical, calm music without lyrics and a soothing calm beat for the use at home (see supplementary material A for the list of music tracks). The CD was a suggestion and participants were also free to choose and listen to other music which they found relaxing. After the instructions were clear to everyone, the group of women heard some examples of the CD and practiced to relax. At the end of the session, the participants were asked to give a second saliva sample and to fill out the questionnaire about subjective well-being (SAM).

The singing group joined approximately two singing sessions between the first time of measurement (T1) and the 34th gestational week. Up to seven women were able to participate in one intervention session that took place weekly at 13:00 h in the same room as the music intervention took place in. The procedure of the singing session was identical to the music session, only that the intervention itself was different. A music therapist gave instructions and practiced some children´s songs and lullabies together with the group that was accompanied by live guitar play. After arriving, the women were asked to give a saliva sample and to fill out the first questionnaire about their subjective well-being (SAM). All participants were then invited to sit down together on gymnastic balls and to make themselves comfortable. The music therapist explained the goal of the intervention—to sing for the own relaxation and interact with the unborn fetus—and gave some detailed information about how to perform the intervention at home. It was highlighted that it is important to perform the singing intervention without any disturbances if possible and it was emphasized that it would be beneficial to integrate the intervention as daily routine. Participants were asked to continue the intervention on a daily basis for 10–15 min per day and to sing children´s songs and lullabies at home until birth. The importance of the mother´s voice was explained as well as the possibility to hum instead of singing. All participants received a song book with lyrics and melodies of ten children´s songs and lullabies (see supplementary material B) as a suggestion for the use at home. The music therapist practised all songs from the song book together with the participants and accompanied the session with live guitar playing. When no unanswered questions were left, the participants insalivated a second saliva sample and filled out the questionnaire (SAM) for the second time. Participants who took part for a second (or third time) were not asked to give saliva samples but filled out a questionnaire about the frequency of use and pleasure of the intervention at home.

### Procedure

Expectant women were offered participation at the time of birth registration in the clinic around the 30th week of gestation. After the participants gave their informed written consent, the first measurement (T1) took place for which the STAI (state and trait version), EPDS, MAAS, ASKU and VAS (perceived closeness to the baby) were surveyed. Afterwards they were randomized into one of the three study groups (music, singing, control group). Participants of the music and singing group then made an appointment for the first intervention session. The music group participated in the music session once whereas the singing group participated between two and four times in the singing intervention. Before and after the first intervention session, saliva samples were taken and the emotional state was measured with the SAM.

The second measurement (T2) was an online questionnaire. At the beginning of the 36th gestational week, the participants received an email with a link to the online platform of SoSci Survey [47] and were asked to complete the same questionnaires that were given at T1 within the next 3 days. When they did not fill out the questionnaires within the stated period, they received up to two reminders via e-mail or by telephone. In order to control the use of music and singing and the realization of the interventions, participants were asked if they listened to music and if they sang for the baby on a regularly basis.

Two further measurement points were part of the larger project which should be mentioned here but are not considered for the present research question as the present paper concentrates on the effects *during* pregnancy. Within the first 48 h after childbirth the third measurement (T3) took place. Participants were asked to fill in questionnaires about the maternal well-being; mother–infant bonding and depressive symptoms using the paper–pencil method and medical information (e.g. birthmode, gestational age, duration of birth) were taken from the medical records. At this time point the participants received a birth present. The fourth and last measurement (T4) took place 8 weeks postpartum and was an online questionnaire with the mentioned questionnaires from T3. After completing the last time of measurement, participants received a present as a “thank you” for their participation per post.

### Statistical analysis

For statistical analysis, the statistical software package SPSS 24 (IBM Inc., Armonk, NY) was used. For the EPDS and MAAS, up to two missing values were conservatively replaced by sample mean scores for respective items [49]. Missing values of the STAI were replaced by mean scores of the norm sample as suggested by Laux et al. [46] when less than two items were missing.

Pre-intervention group-differences were checked by comparing general anxiety (STAI Trait), age and gestational age with univariate ANOVAs. To check for group differences regarding parity, a chi-square test was used. Furthermore, in order to check whether the dependent variables were normally distributed, Shapiro–Wilk tests were calculated.

In order to explore the immediate effects of music and singing 2 × 2 mixed-factorial ANOVAs with the between-subject factor *group* (music and singing) and the within-subject factor *time of measurement* (pre and post intervention comparison) were calculated. All factors of SAM (valence, arousal and dominance) as well as salivary cortisol, alpha-amylase and oxytocin levels were used as dependent variables for the pre-post-calculations. In order to disentangle possible significant interactions post-hoc comparisons with independent *t*-tests were conducted with the amount of alternations (difference-scores) as the dependent variable. Additionally dependent *t*-tests were calculated with the scores of T1 and T2 to investigate the strength of alternations within groups.

To investigate the short-term effects of music and singing 3 × 2 mixed-factorial ANOVAs with the between-subject factor *group* (music, singing and control) and the within-subject factor *time of measurement* (T1 and T2) were calculated. The VAS (closeness to the baby), MAAS (subscores quality and intensity), EPDS and general self-efficacy score were used as the dependent variable respectively. If significant interaction effects were revealed, post-hoc comparisons were conducted to explore group differences in the amount of alternation. Furthermore, within-subject *t*-tests were calculated between times of measurement to explore the amount of alternation in detail.

## Results

The final sample of the MUSICA study consisted of 172 pregnant women. At baseline (T1) the three groups did not differ regarding parity and trait anxiety (*p* ≥ 0.137). However, the difference between groups regarding the maternal age at the first time of measurement was significant [*F*(2,153) = 4.05, *p* = 0.019, *d* = 1.13]. Bonferroni corrected post-hoc comparisons showed a significant difference between the singing and the control group (*p* = 0.018) but not for the other two group comparisons (*p* ≥ 0.151).^1^ The group difference regarding the gestational age was also significant [*F*(2,169) = 5.99, *p* = 0.003, *d* = 0.14]. Bonferroni corrected post-hoc comparisons revealed a significant difference between the singing and the control group (*p* = 0.002), but not for the other groups (*p ≥ *0.114)^1^. For an overview of the group characteristics at T1 and the test-statistics see Table 1. Shapiro–Wilk tests revealed that some variables are not normally distributed (*p* < 0.05) but due to the robustness of ANOVAs (Blanca et al. 2017; Schmider et al. 2010) and the absence of non-parametric alternatives for repeated-measures ANOVAs, all calculations were conducted as intended.

**Table 1:** Sample characteristics—descriptive statistics (means (standard deviations)) and results (*p* values) of univariate ANOVAs at the first time of measurement (T1)

	Music group	Singing group	Control group	*p* value
Sample	*n* = 64	*n* = 59	*n* = 49	
Age (in years at T1)*	34.26 (4.14)	32.81 (3.29)	35.00 (3.85)	*p* = .019^a^
Gestational age (T1)*	31.83 (3.35)	30.63 (3.67)	32.72 (2.15)	*P* = .003^a^
Primipara*	*n* = 37	*n* = 40	*n* = 23	
Multipara*	*n* = 26	*n* = 17	*n* = 22	*p* = .137^b^
STAI Trait	46.25 (5.80)	45.77 (3.54)	45.33 (4.89)	*p* = .605^a^

^a^Result of an univariate ANOVA

^b^Result of a *χ*^*2*^ test

^*^Missing values (*n* = 7)

### Immediate effects—subjective measurements

**Fig. 2:**
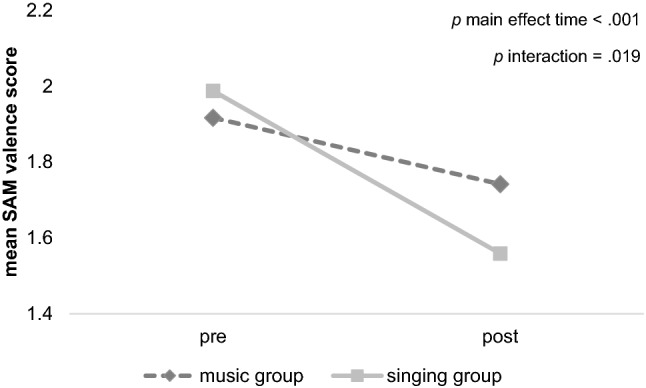
Development of SAM valence scores from pre to post intervention. Both groups show a decrease from pre to post (*p* < .001). The singing group shows a larger reduction of the mean SAM valence score indicating a larger improvement in subjective perceived pleasure than the music group

Regarding the variable SAM valence, a reduction of the score (indicating an improvement of valence) was visible from pre to post intervention for both groups with a larger reduction in the singing group compared to the music group (see Table 2). A mixed-factorial ANOVA with the dependent variable SAM Valence showed a significant main effect for the factor *time of measurement* [*F*(1,117) = 31.76, *p* < 0.001, *d* = 1.13]. While the main effect for the factor *group* was not significant [*F*(1,117) = 0.23, *p* = 0.634, *d* = 0.14], the interaction effect was also significant [*F*(1,117) = 5.61, *p* = 0.019, *d* = 0.47]. In order to explore the interaction effect, post-hoc comparisons of the alternations between groups were conducted with a *t*-test for independent samples. The post-hoc explorations showed a significant difference between the singing and the music group [*t*(106.412) = 2.50, *p* = 0.014, *d* = 0.47] with a larger improvement in the singing group (*M* = − 0.45, *SD* = 0.63) compared to the music group (*M* = − 0.18, *SD* = 0.52). Within-subject *t*-tests for dependent samples showed a significant alternation from pre to post intervention in the singing group [*t*(55) = 5.35, *p* < 0.001, *d* = 0.71] as well as in the music group [*t*(57) = 2.66, *p* = 0.010, *d* = 0.24]. See Fig. 2 for an illustration and an overview of the results of SAM valence. See Table 2 for the extensive descriptive statistics of the immediate effects from pre to post intervention.

**Table 2:** Descriptive statistics (means (standard deviations)) of the variables that were measured pre and post intervention (at the start and immediately after the intervention) showing the immediate effects of the intervention for both groups^a^

	Time of measurement	Music group	Singing group
SAM valence	Pre	1.92 (0.80)	1.99 (0.62)
	Post	1.74 (0.68)	1.56 (0.68)
SAM arousal	Pre	3.75 (0.86)	3.55 (0.86)
	Post	4.25 (0.66)	4.94 (5.36)
SAM dominance	Pre	3.77 (0.76)	3.68 (0.71)
	Post	3.98 (0.71)	3.95 (0.83)
Salivary cortisol (in nmol/l)	Pre	6.39 (2.73)	8.17 (3.85)
	Post	5.82 (2.46)	6.66 (2.62)
Salivary alpha-amylase (in U/ml)	Pre	177.34 (180.78)	172.45 (183.68)
	Post	162.37 (109.72)	156.37 (149.34)
Salivary oxytocin (in pg/ml)	Pre	1.04 (0.23)	1.00 (0.29)
	Post	1.11 (0.33)	1.04 (0.29)

^a^The listed variables are not available for the control group because the control group did not participate in interventions

For the subscore SAM arousal, an improvement occurred from pre to post intervention (see Table 2). A main effect of the factor *time of measurement* [*F*(1,117) = 7.49, *p* = 0.007, *d* = 0.51] was revealed indicating a significant lower arousal at the end of the intervention. The main effect for the factor *group* [*F*(1,117) = 0.42, *p* = 0.516, *d* = 0.13] and the interaction [*F*(1,117) = 1.71, *p* = 0.194, *d* = 0.25] were non-significant.

In both groups, the score of SAM dominance increased from pre to post intervention (see Table 2) which indicates a higher perceived independence at the end of the intervention. For the subscore SAM dominance, a significant main effect for the factor *time of measurement* [*F*(1,115) = 19.27, *p* < 0.001, *d* = 0.77] was revealed but neither the effect of the factor *group* was significant [*F*(1,115) = 0.20, *p* = 0.652, *d* = 0.06], nor was the interaction [*F*(1,115) = 0.06, *p* = 0.553, *d* = 0.06].

### Immediate effects—physiological measurements

**Fig. 3:**
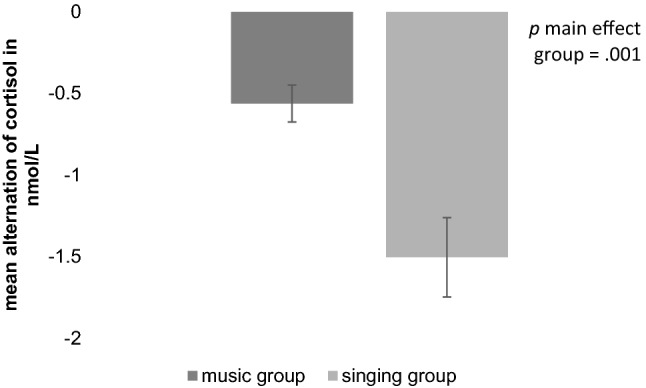
Amount of the alternation of salivary cortisol in the music and singing group. Pictured are the mean difference-scores from pre to post intervention. The singing group shows a larger stress reduction visible through a larger reduction of salivary cortisol indicating less stress after the intervention (*p* = .001)

For the level of salivary cortisol, a decrease during the intervention occurred in both groups and furthermore the singing group showed a larger reduction than the music group (see Table 2). Significant main effects for the factors *time of measurement* [*F*(1,114) = 61.83, *p* < 0.001, *d* = 1.43] and *group* [*F*(1,114) = 6.02, *p* = 0.016, *d* = 0.42] were found. In addition, a significant interaction [*F*(1,114) = 12.02, *p* = 0.001, *d* = 0.65] was revealed. A post-hoc comparison regarding the alternation from pre to post intervention showed a significant difference between groups [*t*(76.932) = 3.40, *p* = 0.001, *d* = 0.65]. The singing group had a larger reduction of cortisol (*M* = − 1.53 nmol/l, *SD* = 1.91) than the music group (*M* = − 0.58 nmol/l, *SD* = 0.86; see Fig. 3). Within group comparisons with dependent *t*-tests revealed a significant reduction in the singing group [*t*(54) = 4.94, *p* < 0.001, *d* = 0.35] as well as in the music group [*t*(55) = 6.00, *p* < 0.001, *d* = 0.21].

The levels of alpha-amylase were stable during the intervention without a difference between groups (see Table 2). The analysis of alpha-amylase with a mixed-factorial ANOVA showed no significant effects of the factor *time of measurement* [*F*(1,113) = 2.14, *p* = 0.147, *d* = 0.25] or *group* [*F*(1,113) = 0.04, *p* = 0.844, *d* = 0.11]. The interaction [*F*(1,113) = 0.003, *p* = 0.959, *d* = 0.06] was also non-significant. A reduction is only present in a descriptive way (see Table 2).

In regard to the level of salivary oxytocin, both groups showed a significant increase from pre to post intervention (see Table 2). For salivary oxytocin, a significant main effect of the factor *time of measurement* [*F*(1,112) = 6.42, *p* = 0.013, *d* = 0.48] was found. The effects of the factor *group* [*F*(1,112) = 1.21, *p* = 0.274, *d* = 0.21] and the interaction [*F*(1,112) = 0.24, *p* = 0.628, *d* = 0.09] were not significant.

### Short-term effects

**Fig. 4:**
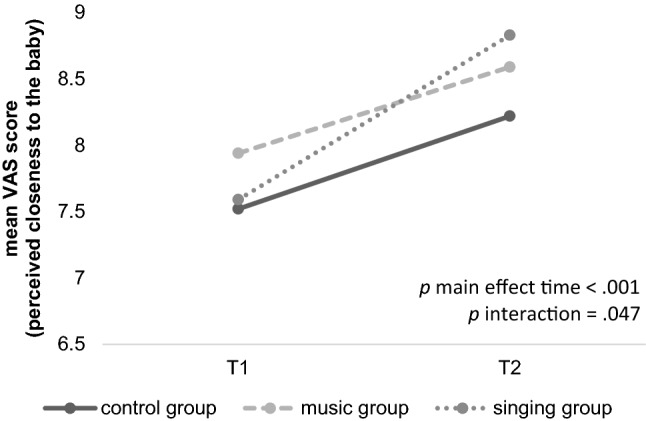
Development of VAS (closeness to the baby) scores from the first to the second time of measurement, pictured for each group separately. All groups reported a higher perceived closeness to the baby at the second time of measurement compared to the first point (*p* < .001). A significant time*group interaction was visible (*p* = .047) as the singing group showed the largest improvement

The calculations for the short term effects for the dependent variable VAS (closeness to the baby) showed an improvement over time and a significant interaction of time and group highlighting different amounts of alternation from T1 to T2 depending on group allocation. (see Table 3). A significant main effect was found for the factor *time of measurement* [*F*(1,138) = 51.15, *p* < 0.001, *d* = 1.33] and no effect was found for the factor *group* [*F*(2,138) = 1.05, *p* = 0.352, *d* = 0.25]. A significant interaction [*F*(2,138) = 3.12, *p* = 0.047, *d* = 0.42] was revealed. Post-hoc comparisons regarding the amount of increase revealed no significant differences between groups but a trend (*p* ≥ 0.065). The alternation in the VAS (closeness to the baby) from T1 to T2 was significant for the control group [*t*(38) = − 3.68, *p* = 0.001, *d* = 0.42] as well as for the music [*t*(52) =—3.61, *p* = 0.001, *d* = 0.46] and the singing group [*t*(48) = -6.37, *p* < 0.001, *d* = 0.77]. The singing group showed the greatest increase of closeness (*M* = 1.23, *SD* = 1.36) compared to the music (*M* = 0.64, *SD* = 1.30) or control group (*M* = 0.70, *SD* = 1.19; see Table 3 and Fig. 4) with the largest effect size.

**Table 3:** Descriptive statistics (means (standard deviations)) and results (*p* values) of univariate ANOVAs measuring group differences for the short-term variables at T1 and T2

	Music group	Singing group	Control group	*p* value
Sample	*n* = 64	*n* = 59	*n* = 49	
VAS Closeness^T1^	8.00 (1.41)	7.58 (1.80)	7.37 (1.76)	*p* = .116
VAS Closeness^T2^	8.59 (1.38)	8.83 (1.17)	8.22 (1.49)	*p* = .114
MAAS Quality^T1^	48.42 (2.91)	47.83 (2.77)	47.57 (3.08)	*p* = .276
MAAS Quality^T2^	51.39 (2.97)	50.67 (4.05)	51.15 (2.90)	*p* = .545
MAAS Intensity^T1^	28.67 (5.14)	29.17 (3.97)	28.33 (4.74)	*p* = .637
MAAS Intensity^T2^	29.52 (5.12)	30.52 (4.38)	29.25 (4.29)	*p* = .369
ASKU^T1^	4.25 (0.57)	4.19 (0.54)	4.18 (0.41)	*p* = .773
ASKU^T2^	4.28 (0.56)	4.31 (0.41)	4.05 (0.33)	*p* = .014 *
EPDS^T1^	5.51 (4.11)	5.10 (3.70)	5.67 (4.85)	*p* = .766
EPDS^T2^	4.92 (3.97)	5.47 (3.84)	4.98 (3.88)	*p* = .651

^T1^first time of measurement (around 30th gestational week)

^T2^second time of measurement (around 36th gestational week)

^*^significant difference between groups (*p* < .05)

In regard to the MAAS subscore *quality* all groups showed a similar improvement from T1 to T2 (see Table 3). The analysis of the MAAS subscore *quality* showed a significant main effect of the factor *time of measurement* [*F*(1,143) = 153.20, *p* < 0.001, *d* = 2.07]. The main effect of the factor *group* [*F*(2,143) = 0.71, *p* = 0.492, *d* = 0.20] and the interaction effect [*F*(2,143) = 0.37, *p* = 0.688, *d* = 0.14] were non-significant. For the subscore *intensity*, a similar increase over time was found for all groups (see Table 3) indicating an increase of bonding intensity. A significant effect of the factor *time of measurement* was revealed [*F*(1,143) = 20.54, *p* < 0.001, *d* = 0.76]. The effect of the factor *group* [*F*(2,143) = 0.82, *p* = 0.442, *d* = 0.21] and the interaction effect [*F*(2,143) = 0.27, *p* = 0.767, *d* = 0.13] was non-significant.

**Fig. 5:**
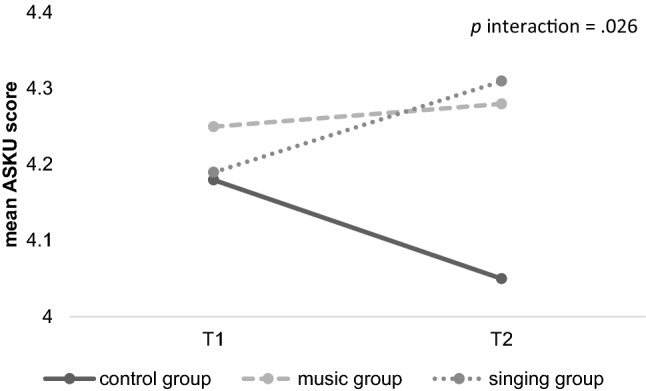
Development of general self-efficacy (ASKU mean scores). A significant interaction between time and group was revealed (*p* = .026). The values in the singing group increased significantly (*p* = .038), whereas the increase for the music group was non-significant and the control group even showed a descriptively decrease in self-efficacy (*p* ≥ .109)

The score of general self-efficacy showed different directions of alternation depending on group (see Table 3). The singing group showed a significant increase of self-efficacy, whereas the alternations in the control and music group were non-significant. No significant main effects of the factor *time of measurement* [*F*(1,141) = 0.07, *p* = 0.798, *d* = 0] or the factor *group* [*F*(2,143) = 1.62, *p* = 0.202, *d* = 0.03] was revealed but the interaction effect was significant [*F*(2,143) = 3.75, *p* = 0.026, *d* = 0.46]. In order to further explore the interaction effect, post-hoc comparisons between groups regarding the alternation were conducted that showed a significant difference between the control and the singing group (*p* = 0.021) but not between the others (*p* > 0.05). Furthermore, within-subject *t*-tests for dependent samples showed a significant improvement over time only for the singing group [*t*(51) = -2.13, *p* = 0.038, *d* = 0.25] but not for the music or the control group (*p* ≥ 0.109; see Table 3 and Fig. 5).

In all three groups, the EPDS score did not change from T1 to T2 and no group effect was revealed (see Table 3). No significant main effects of the factor *time of measurement* [*F*(2,141) = 1.70, *p* = 0.194, *d* = 0.22] or the factor *group* [*F*(2,141) = 0.03, *p* = 0.969, *d* = 0] were found. In addition, the interaction effect [*F*(2,141) = 1.19, *p* = 0.306, *d* = 0.26] was also non-significant.

## Discussion

The present study investigated the effect of a prenatal music and singing intervention on maternal well-being and mother–infant bonding during pregnancy. The study revealed positive immediate effects of active singing and passive music listening on salivary cortisol and oxytocin as well as on valence, arousal and dominance. Short-term effects were revealed on the perceived closeness to the unborn child measured with VAS and self-efficacy. In regard to perceived closeness (VAS), the intervention groups showed a significant larger improvement over time with overall higher values in comparison to the control group. For self-efficacy as well, a significant greater improvement was revealed for the intervention groups compared to the control group. In regards to differences between the two types of intervention, the results highlight larger positive effects in the singing group compared to the music group on valence, cortisol regarding immediate effects as well as short-term effects on perceived closeness to the unborn child and self-efficacy. However, no effects of either intervention on depressive symptoms or the bonding questionnaire could be shown.

Regarding immediate effects, the interventions showed positive effects on the emotional state (valence, arousal and dominance) of the mother-to-be. The women reported a greater feeling of control (dominance), lower valence scores and less excitement e.g. more relaxation after both intervention types. These results are in accordance with previous studies highlighting relaxing and mood improving effects of listening to music and singing during pregnancy [9, 10, 26, 63]. Furthermore, the results regarding the valence scores point in favour of singing. The singing group showed a significant greater improvement in the SAM valence scores with lower values indicating more happiness at the time point post intervention. This is interesting and indicates that the active part of singing may have an additional positive effect on happiness which could maybe be explained by findings that singing leads to an increased release of endorphins which is associated with feelings of happiness [39].

Also, the results of the physiological measurements of salivary cortisol and oxytocin revealed immediate positive effects of music listening and singing. The significant reduction of salivary cortisol levels is in line with other studies which investigated the impact of relaxation techniques on salivary cortisol levels also revealing a decline from before until after the intervention [64, 82]. Moreover, the two groups differed in the amount of reduction with a significant larger improvement of salivary cortisol in the singing group. Singing seems to have a larger stress-reducing impact on the cortisol stress response than listening to music. Although positive effects on salivary cortisol levels have been reported for each kind of musical intervention [18, 64, 82] the present study is the first which made a direct comparison between both, music and singing, in the context of pregnancy. A point to acknowledge is that the intervention of the singing group was earlier in the day than the music group. Therefore influences of the circadian rhythm may occur. Cortisol levels are highest in the morning and decrease during the day [43]. However, the results of the difference-scores which take this into account, revealed that the singing group showed larger reductions in cortisol indicating a larger stress-reducing effect. It would be important for future studies to conduct the interventions at the same time in order to better control the influence of time of day on cortisol levels.

The significant increase of oxytocin in both interventions fits well to previous research [45, 62, 64]. It has been shown that levels of stress are negatively associated with oxytocin [20]. Furthermore, it has already been shown that relaxing music interventions led to higher levels of oxytocin [62, 64]. Beside the association with stress and relaxation, oxytocin also seems to be associated with social interactions. Supportive social interactions cause a raise of oxytocin levels [28, 36] and furthermore, an increase of oxytocin was reported during parent-infant communication [20]. Especially in mother–infant relationships and interactions, oxytocin seems to play an important role [22]. However, contrary to our hypothesis, the active and communicative part of singing seems to have no additional impact on the amount of oxytocin increase. The interplay of an increase of oxytocin in addition to a reduction of cortisol are also in line with reported effects regarding the application of synthetic oxytocin which is associated with a suppression of cortisol and a lower subjective stress response [30]. Overall, the here presented results of cortisol and oxytocin indicate a relaxing effect of the music and singing interventions.

No effects were found for saliva alpha-amylase. The results of saliva alpha-amylase are in contrast to other studies that reported lower levels of alpha-amylase in response to a relaxing music intervention [44, 52]. Usually the response of alpha-amylase is sensitive to stress [40] and decreases as a result of relaxation [16]. In general, the level of alpha-amylase is modulated by the activity of the sympathetic nervous system and increases in response to acute stress [58]. It is possible that singing leads to a physiologic activation which in turn activates the sympathetic system [25]. One could argue that this activation counteracts the relaxation process which is visible in the missing effects regarding response of alpha-amylase to the music and singing interventions. However, missing effects of music interventions on salivary alpha-amylase have also been reported previously [32].

Aside from the immediate effects, also positive short-term effects of the interventions on certain variables could be shown. The perceived closeness to the baby measured by VAS and the Maternal Antenatal Attachment Scale (MAAS) increased over time from the first (recruitment) to the second (36th gestation week) time of measurement which is in line with other studies that show an increase of bonding towards the end of pregnancy [68]. Higher scores can be caused by growing fetal feedback during the last trimester [55]. The analysis of the present study also revealed a significant interaction effect for the VAS. Even though the post-hoc analysis revealed non-significant results the difference scores highlight that the singing group showed a substantial larger increase of perceived closeness compared to the music and control group. Although a significant interaction effect was not present in the MAAS, the positive effect of singing on perceived closeness measured by VAS indicates that the active part of singing to the unborn baby can positively influence bonding between mother and child which is in line with other studies [9, 17]. The here presented indications of improved prenatal bonding as a result of singing during pregnancy is in contrast to Persico et al. [67] who only reported effects on postnatal bonding. Although we acknowledge that the post-hoc tests did not turn out significant, we believe that the significant interaction effect, which indicates a different amount of improvement regarding perceived closeness measured with VAS between groups, is interesting and meaningful. The difference scores highlight that the singing group shows the greatest improvement, which in turn underlines that the singing intervention has a more positive impact on mother-infant bonding than the music intervention. The greater benefit of singing in comparison with a music intervention regarding bonding is in line with the results of Fancourt and Perkins [17] who reported a positive impact on attachment through singing but not through music interventions in a postnatal context. It has to be noted that the few studies on the effects of musical interventions on bonding during pregnancy are difficult to compare because context, gestational age and frequency of interventions varies over studies.

Additionally, the present study showed that the two intervention groups showed an increase in self-efficacy which was significantly larger in the singing group. In this regard, Sun et al. [78] discovered that a prenatal yoga programme as a learned active coping strategy for emotional regulation can also lead to an improved self-efficacy. Overall, high self-efficacy seems to have several stress-buffering effects and impact on the physiological as well as on the psychological state [73]. The effect of learned relaxation seems to be one reason for higher self-efficacy [85]. As studies have shown that higher self-efficacy scores lead to reduced physiological stress-reactions during pregnancy [61], to improved coping capacities during labour [23, 38] and to a better identification with the motherhood [38], the here reported positive effects of music and especially singing should encourage further research. It would be desirable to investigate whether the positive effects maintain over a longer time period.

Regarding the influence of the interventions on depressive symptoms, the results show no significant effects. Contrary to our hypothesis and results of previous studies [8, 10], no interaction between time and group was revealed. Overall in the present study, all participating women had rather low EPDS scores (*M = *5.42, *SD* = 4.19) at the time of recruitment (T1) and only 12 of them reached the cut-off score of 13 which indicates a higher probability to suffer from depression [14]. Therefore, it can be hypothesised that an improvement of depressive symptoms could not be detected as the variance and potential to improve was too low. In further studies it would be interesting to investigate the effects of a singing intervention in a high-risk sample, for example women who suffer from prenatal depression. In this regard, Perkins, Yorke and Fancourt [66] conducted a study with mothers who suffered from postnatal depression and discovered with qualitative interviews that singing can reduce symptoms of postnatal depression.

A number of limitations of the study warrant a comment here. Unfortunately, not all women filled in the questionnaires completely that led to missing values which in turn may have biased the results. Although, we put a lot of effort in reminding the participants to fill in the questionnaires, the generalization is limited as for example we cannot rule out that maybe women that were extremely stressed or may display higher depressive symptoms did not take part at the second measurement point which may have biased the results. However, a systematic drop out or missing values depending on group allocation can be omitted. Furthermore, although participants were asked at the second time of measurement whether they listened to music or sang for themselves and the baby in order to control whether the women complied to the instructions to perform the interventions at home on a daily basis, the answers are only self-reported and the at-home interventions are therefore not standardized and well controlled. Moreover, also a selection bias cannot be ruled out as many women also denied participation when offered to take part. Some women did not take part as they were afraid to be randomised into the singing group as they expressed not to be comfortable with singing. As a result, it is possible that only pregnant women who were not averse to music or singing took part in the study. In addition, we acknowledge that significant although subtle group differences were revealed regarding age and gestational week at T1. Additional calculations with age and gestational week at T1 as covariates revealed no statistical influence on the reported effects. Therefore, we would argue that these differences are not meaningful in regards to the addressed research question.

In the present study, a number of promising results of passive music listening and active singing interventions during pregnancy were revealed. Immediate positive effects could be shown on the emotional state (valence, arousal and dominance) and on salivary cortisol as well as on oxytocin with significant larger effects in the singing group for valence and cortisol. Furthermore short-term effects regarding general self-efficacy and perceived closeness to the child measured by VAS were revealed for which the improvements were larger in the singing group compared to the music group and control group. The applied interventions could be a simple, cost-effective method to reduce stress and improve well-being of the mother-to-be.

## Electronic supplementary material

Below is the link to the electronic supplementary material.

Supplementary file1 (PDF 22 kb)

## Footnotes

1 Additional calculations regarding group differences were performed with the variables maternal age and gestational age as covariates. For all dependent parameters no significant influence was revealed.
