# Supplementary material for: The effects of a music and singing intervention during pregnancy on maternal well-being and mother–infant bonding: a randomised, controlled study
Source: Arch Gynecol Obstet. 2020 Aug 10;303(1):69–83. doi: 10.1007/s00404-020-05727-8 (PMC7854426; doi:10.1007/s00404-020-05727-8)
Supplement: Supplementary file 1 — Supplementary file1 (PDF 22 kb) [file 404_2020_5727_MOESM1_ESM.pdf]

Supplementary Material

Article title: The effects of a music and singing intervention during pregnancy on maternal well-being and mother-infant bonding: a randomised, controlled study

Journal name: Archives of Women´s Mental Health

Author names: Verena Wulff[1], Philip Hepp[2, 3], Oliver T. Wolf[4], Percy Balan[5], Carsten Hagenbeck[5], Tanja Fehm[5] & Nora K. Schaal[1]

Affiliation:

[1]Department of Experimental Psychology, Heinrich-Heine-University Düsseldorf, Düsseldorf, Germany
[2]Clinic for Gynecology and Obstetrics, University Clinic, Augsburg, Germany
[3]Clinic for Gynecology and Obstetrics, HELIOS University Clinic, Wuppertal, University Witten/Herdecke, Germany
[4]Department of Cognitive Psychology, Institute of Cognitive Neuroscience, Faculty of Psychology, Ruhr-University Bochum, Germany
[5]Clinic for Gynecology and Obstetrics, Heinrich-Heine-University Düsseldorf, Düsseldorf, Germany

E-mail address of the corresponding author: verena.wulff@hhu.de

Material A

List of songs (CD for the music group)

Number	Title	Author
1	River flows in you	Yiruma
2	Sinfonia Concertante for Four Winds in E-flat major, K. 297b	Wolfgang Amadeus Mozart
3	Pachelbel´s Canon (Canon and Gigue for 3 violins and basso continuo)	Johann Pachelbel
4	Serenade No. 13 for strings in G major („Eine kleine Nachtmusik“)	Wolfgang Amadeus Mozart
5	Concerto No. 4 in F minor („Winter“), II. Largo	Antonio Vivaldi
6	Le Carnaval des animaux	Camille Saint-Sans
7	Nocturne in E-flat major, Op. 9, No. 2	Frédéric Chopin
8	"Air" from Bach's Orchestral Suite No. 3	Johann Sebastian Bach
9	Piano Concerto No. 21 in C major, II. Andante	Wolfgang Amadeus Mozart
10	Violin Concerto No. 3 in G major, II. Adagio	Wolfgang Amadeus Mozart
11	Le Onde	Ludovico Einaudi
12	Peer-Gynt Suite Nr. 1, Morning Mood	Edvard Grieg
13	Suite bergamasque, III. Claire de lune	Claude Debussy
14	Clarinet Concerto in A major, II. Adagio	Wolfgang Amadeus Mozart

Material B

List of songs (Children´s songs and lullabies for the singing group)

Number	Original (German) Title
1	La-Le-Lu
2	Der Mond ist aufgegangen
3	Schlaf, Kindlein schlaf
4	Stille, Stille, kein Geräusch gemacht
5	Twinkle twinkle, little star
6	Kommt ein Vogel geflogen
7	Taler, Taler, du musst wandern
8	Summ, summ, sum
9	Die Blümelein, sie schlafen
10	Bruder Jakob
